# Integrated Analysis of Osmotic Stress and Infrared Thermal Imaging for the Selection of Resilient Rice Under Water Scarcity

**DOI:** 10.3389/fpls.2022.834520

**Published:** 2022-02-14

**Authors:** Naima Mahreen, Sumera Yasmin, M. Asif, Sumaira Yousaf, Mahreen Yahya, Khansa Ejaz, Hafiz Shahid Hussain, Zahid Iqbal Sajjid, Muhammad Arif

**Affiliations:** ^1^Soil and Environmental Biotechnology Division, National Institute for Biotechnology and Genetic Engineering College, Pakistan Institute of Engineering and Applied Sciences (NIBGE-C, PIEAS), Faisalabad, Pakistan; ^2^Agricultural Biotechnology Division, National Institute for Biotechnology and Genetic Engineering College, Pakistan Institute of Engineering and Applied Sciences (NIBGE-C, PIEAS), Faisalabad, Pakistan; ^3^Nuclear Institute for Agriculture and Biology College, Pakistan Institute of Engineering and Applied Sciences (NIAB-C, PIEAS), Faisalabad, Pakistan

**Keywords:** osmotic stress tolerance, plant temperature, water deficit, infrared thermal imaging, proline

## Abstract

The climate change scenario has increased the severity and frequency of drought stress, which limits the growth and yield of rice worldwide. There is a dire need to select drought-tolerant rice varieties to sustain crop production under water scarcity. Therefore, the present study effectively combined morpho-physiological and biochemical approaches with the technology of infrared thermal imaging (IRTI) for a reliable selection of drought-tolerant genotypes. Initially, we studied 28 rice genotypes including 26 advance lines and three varieties for water stress tolerance under net house conditions. Three genotypes NIBGE-DT-02, KSK-133, and NIBGE-DT-11 were selected based on the Standard Evaluation System (SES) scoring for drought tolerance. NIBGE-DT-02 showed tolerance to polyethylene glycol (20%) induced osmotic stress indicated by a minimum reduction in seedling length, biomass, chlorophyll content, and increased leaf proline content as compared to susceptible varieties under a hydroponic system. NIBGE-DT-02 was further evaluated for water withholding at varying growth stages, i.e., 30 and 60 days after transplantation (DAT) in pots under net house conditions. NIBGE-DT-02 showed a significantly lower reduction (35.9%) in yield as compared to a susceptible variety (78.06%) under water stress at 60 DAT with concomitant induction of antioxidant enzymes such as peroxidase, catalase, and polyphenol oxidase. A significant increase (45.9%) in proline content, a low increase (7.5%) in plant temperature, along with a low reduction in relative water content (RWC) (5.5%), and membrane stability index (MSI) (9%) were observed under water stress at 60 DAT as compared to the well-watered control. Pearson correlation analysis showed the strong correlation of shoot length with MSI and root length with RWC in rice genotypes at the later growth stage. Furthermore, Regression analysis indicated a negative correlation between plant temperature of NIBGE-DT-02 and proline, RWC, MSI, and peroxidase enzyme under variable water stress conditions. All these responses collectively validated the adaptive response of selected genotypes under water stress during different growth stages. Tolerant genotypes can be used in breeding programs aimed at improving drought tolerance and can expand rice cultivation. Furthermore, this study provides a foundation for future research directed to utilize IRTI as a fast and non-destructive approach for the selection of potent rice genotypes better adapted to water scarcity from wide germplasm collection.

## Introduction

Rice (*Oryza sativa* L.) is an important staple food consumed by more than half of the global human population. It plays a predominant role by providing 50–80% of the daily calories (Fukagawa and Ziska, 2019). It is estimated that annually, 520 million metric tons of rice grains (milled) are produced worldwide (FAOSTAT, 2021) while its grain yield is ≈ 2,562 Kg/ha in Pakistan (Suleri and Iqbal, 2019). In the recent era, climate change is a global problem because many countries around the world are becoming more vulnerable to natural disasters (Tan et al., 2021). As agriculture depends on climate cycles and weather patterns, climate change has caused negative effects on crop productivity and economic returns from the agricultural land (Kavadia et al., 2020).

Rice production has significantly been exposed to a number of abiotic stresses like drought, flood, high temperature, salinity, and heavy metals due to global climate change (Yadav et al., 2020). Among different abiotic stresses, drought is the most challenging as it reduces up to 70% of the rice production globally (Lum et al., 2014). Water scarcity affected one-third of the world’s total rice cultivated area (Kumbhar et al., 2015). The severity of drought is very complex and depends on various causes such as the frequency of rainfall, evaporation, and soil moisture (Oladosu et al., 2019).

Many studies have been reported water stress causing changes in plants’ physiological and biochemical responses, i.e., effects on mineral nutrition, transpiration rate, plant water relations, enzymatic activities, rate of photosynthesis, pigment degradation, stomatal conductance, and process of grain filling (Anjum et al., 2017). In water stress conditions, induction of reactive oxygen species (ROS) stimulates in plants causing DNA mutation, cellular oxidative damages, peroxidation of lipids, and protein denaturation (Sgherri et al., 1996; Hasanuzzaman et al., 2020). Water scarcity is linked with oxidative damage and mechanical interruption during the penetration of plant roots in drought-stressed hard soils. Collectively, these factors affect morpho-physiological and biochemical attributes of plants that ultimately result in reduced crop yield (Wilkinson and Davies, 2010).

Responses of rice genotypes to water stress are complex and widely varied with stress duration, growth stage, and type of genotype. It has been documented that flowering is delayed if water stress occurred between panicle initiation and pollen meiosis due to the delay in the development of flowers and other parts (Ji et al., 2012). Many studies have been reported that water scarcity in rice is more sensitive for seedling and booting/flowering stages (Sridevi and Chellamuthu, 2015). Less reduction in the yield of rice is observed if water stress occurs at a vegetative stage but the same stress can cause a severe yield decrease if it occurs at the time of fertilization (Raza et al., 2019). The water stress tolerance of plants at the initial stages of development is of prime importance, because good seed germination and better growth of seedlings under water deficit conditions may show potential tolerance at later growth stages to attain higher yields (Sun et al., 2020).

Rice is a water-demanding crop during the irrigated ecosystem. It needs about three to five thousand liters of water for irrigation to harvest 1 kg paddy rice in Pakistan (Sabar and Arif, 2014). To overcome the adverse effects of climate change on water availability, there is a prime need for the development or identification of suitable rice genotypes tolerant to water scarcity that can give sustainable yield under water-stressed conditions (Fahad et al., 2017). In most of the studies for screening tolerance to drought, water deficit condition is usually induced either by (1) withholding irrigation to grown plants in fields or soil pots in greenhouse experiments covered with shelter or (2) elevated the osmotic potential for grown plants in hydroponics with variable osmoticums, e.g., polyethylene glycol (PEG) (Cai et al., 2020).

A better understanding of rice morphological, biochemical, and molecular mechanisms involved in tolerance to water scarcity is extremely important to improve the rice genotypes (Panda et al., 2021). The standard evaluation scoring system (SES) was used as a primary criterion to screen rice genotypes for water stress tolerance or susceptibility (International Rice Research Institute [IRRI], 2014). Several morpho-physiological parameters, for example, root and shoot lengths, fresh and dry weights, relative water content, membrane stability index (MSI), chlorophyll, and proline content are being used for screening the genotypes for water stress tolerance (Lekshmy et al., 2021). A complex antioxidant system including both enzymatic and non-enzymatic antioxidants is a protection mechanism adopted by plants to avoid the damaging effects of ROS (Hasanuzzaman et al., 2020).

Plant physiological and biochemical attributes for screening drought tolerance and susceptibility are complex, labor-demanding, and time-consuming. Infrared thermal imaging (IRTI) emerged as a promising, user-friendly, and non-destructive technique to measure crop physiological status related to water availability (Wedeking et al., 2016). Imaging-guided expert systems have been recently used in agriculture to analyze the response of different crops (soybean, maize, lentil, and rice) to biotic and abiotic stresses (Pineda et al., 2021). Infrared thermography is a high throughput facility that may contribute to the precise selection of next-generation rice crops to combat adverse climate conditions (Kim et al., 2020).

Keeping in view the importance of reliable *in situ* method for the selection of drought-tolerant superior rice genotypes from a wide collection, the present research was conducted to screen rice genotypes for water stress tolerance ability under PEG-induced osmotic stress at the seedling level in hydroponic nutrient solution and to study the yield components of the selected genotype by imposing water stress (water withholding for 15 days) at early and later growth stages, respectively, in pot experiments. Therefore, the preliminary objective of the present study was to integrate morphological, physiological, and biochemical screening approaches with improved *in situ* methods, i.e., infrared thermal imaging for precise selection of drought-tolerant rice genotypes. Secondly, the objective was to evaluate the selected genotypes under water deficit conditions at different growth stages to ensure their tolerance throughout the whole crop season. The results obtained will be employed for the improvement of the rice crop in future breeding programs to address the food security issues in this alarming situation of climate change.

## Materials and Methods

### Collection of Plant Material Used

A total of twenty-eight rice genotypes including one tolerant (IR-55419-04), one susceptible (Super Basmati) check variety, and 26 advance lines were used for screening their drought tolerance (Supplementary Table 1). Among these, NIBGE-DT-02 and NIBGE-DT-11 were the offspring of Super Basmati as drought-susceptible and IR-55419-04 as drought-tolerant parents (Mumtaz et al., 2019; Sabar et al., 2019). The NIBGE-DB lines were the offspring of NIBGE-BR18 as the drought-susceptible and NIBGE-DT-02 as the drought-tolerant parent. Healthy seeds of 28 genotypes were obtained from Agricultural Biotechnology Division (ABD), National Institute for Biotechnology and Genetic Engineering (NIBGE) Faisalabad.

### Initial Screening of Rice Genotypes in a Pot Experiment Under Rainout Zone

The experiment was conducted under natural net house conditions at NIBGE (11° 26′ N 73°16′ E) during the rice-growing season (July-October, 2019). The nurseries of 28 genotypes were sown. For the healthy seedlings, recommended management practices were done (Zahid et al., 2020). Earthen pots (12-inch diameter, 14-inch heigh) were filled with homogenized 12 kg soil (textured loamy, non-sterilized, EC 3.1 mS cm^–1^, and pH 6.45). The pots were water-saturated for a few days to settle down the soil before transplanting the rice seedlings. The level of the soil 5 cm below the edge of pots was set aside. The experiment with three replicates was laid out in randomized completely block design (RCBD). The experimental pots were then divided into two groups, i.e., well-irrigated (control) and 15 days water-stressed. Seedlings of thirty-five days were transplanted in pots. The recommended dose of nitrogen (N) half and phosphorous (P) full in the form of urea and diammonium phosphate (DAP) were applied at a rate of 50 and 150 mg/kg of the soil, respectively, before transplantation. The remaining N dose was applied after 15 days of transplantation. The pots of control treatments were normally irrigated for the whole duration of the experiment while pots of stress treatment were watered for the first 30 days after transplantation (DAT) and then subjected to water stress (pots without watering) for the next 15 days. Water stress-treated pots were protected from rain during the stress period, i.e., 15 days, and re-irrigated after stress imposition.

After 15 days of water stress, a modified standard evaluation system (SES) scoring of rice genotypes at the seedling stage (Supplementary Table 2) was used to evaluate stress symptoms on leaves. This scoring differentiates the tolerant, moderately tolerant, and susceptible genotypes according to their ability to tolerate water scarcity (International Rice Research Institute [IRRI], 2014).

### Screening of Osmotic Stress Tolerance at Seedling Stage Under Hydroponic Conditions

Three genotypes on the basis of their phenotypic response were selected and a drought tolerance score was observed in their initial screening in pots under net house conditions. These genotypes (NIBGE-DT-02, NIBGE-DT-11, and KSK-133) were further screened under control and polyethylene glycol (PEG) induced osmotic stress hydroponic conditions (Supplementary Table 3). The experiment was carried out in a growth room maintained with 16 h day length, 28°C day, and 23°C night with 460 μ mol/m2/s light intensity. The seeds were surface sterilized with 2% NaOHCl for 5 min, washed thrice with sterilized distilled water then soaked for 3 h in sterilized distilled water to get proper germination. Seeds were placed at an equal distance from each other on filter paper already moistened with sterilized distilled water in Petri plates for germination. The Petri plates were kept in a growth room for 4 days.

Uniform seedlings with good germination were transferred into 96-hole containing seedling boxes filled with sterilized distilled water of the same volume. Twenty-four germinated seedlings of each genotype were placed in 12 holes (two seeds in each hole) in three boxes, separately. All the boxes were labeled and placed on racks supplemented with sterilized water in a growth room for 4 days. Rice seedlings were then shifted to one-fourth of Hoagland solution (Gómez-Luciano et al., 2012) for the next 4 days. In each box, the same level of the nutrient solution was maintained by adding the solution. To decipher the variation in genotypes for drought tolerance, 8 days post-transplantation (DPT) rice seedlings were then subjected to drought stress. Drought stress was imposed by elevating the osmotic potential (PEG-simulated drought) in boxes by adding 5% PEG-8,000 in one-fourth Hoagland solution. The plants grown only in Hoagland solution of one-fourth concentration were considered as a control. Then osmotic potential elevated to 10, 15, and 20%, respectively. The duration for each stress level was 4 days. The experiment was conducted in a complete randomized design (CRD) with three replicates. Afterward, the twenty-eight-day-old plants along with the roots were taken from the boxes to study different morphological traits, i.e., root/shoot lengths, fresh and dry weights. The plants were frozen for further analysis, i.e., different physio-biochemical parameters.

#### Morphological Parameters

The morphological parameters of rice seedlings were studied at the end of the 20% PEG (−0.5 MPa)-simulated osmotic stress. Three plants from each replicate of the control and stressed were collected randomly and studied root/shoot lengths and fresh weights. After recording the fresh weights, the roots and shoots were kept in the oven at 70°C for 48 h to measure their respective dry weights (at 14% water content) (Wimberly, 1983).

#### Physiological Parameters

After the morphological analysis, physiological responses in plant leaves were analyzed. Ten leaves per replicate of the control and stressed plants were collected and chopped to make a composite sample (Motaleb et al., 2018).

##### Leaf Chlorophyll Content

The amount of chlorophyll a, b, and t (total chlorophyll) was calculated in the leaves of the control and stressed plants using the methods described by Arnon (1949) and Davies (1976). Leaf sample (0.1 g) was ground in 80% chilled acetone and incubated at 10°C for 24 h. After 24 h, the sample was centrifuged at 14,000 × *g* for 5 min. The absorbance of the supernatant was measured at 470,645, and 663 nm.

##### Leaf Proline Content

Proline content (μmolg^–1^ fresh weight) was measured with the method described by Bates et al. (1973). A leaf sample (0.5 g) from a homogenized sample of each replicate was ground in 3% 5-Sulfosalicylic acid, followed by centrifugation at 13,000 rpm for 10 min. The supernatant (2 mL) was added to a test tube with 2 mL of acid ninhydrin, 2 mL of glacial acetic acid, and 2 mL of 6 M phosphoric acid then incubated in a water bath at 100°C for 1 h. After 1 h, the reaction mixture was then cooled in an ice bath for 10 min. Toluene (4 mL) was added to the reaction mixture and mixed vigorously with a test tube stirrer for 20–30 s. The organic toluene phase (upper layer) containing the chromophore was collected and the absorbance of pink to red color developed was estimated at 520 nm using a spectrophotometer (Carry60 UV-Vis, Agilent Technologies, United States). Proline concentration was determined using a standard curve developed with different concentrations of L-proline (Bates et al., 1973).

### Validation of Tolerant Rice Genotype for Drought Tolerance in a Pot Experiment Under Rainout Zone

Based on the findings from both the initial screening in a pot experiment followed by hydroponically PEG-simulated screening for drought tolerance, the rice genotype (NIBGE-DT-02) was selected for further validation in a pot experiment not only for their tolerance to water scarcity but also for physio-biochemical and yield parameters. The experiment was conducted under natural net house conditions at NIBGE (11° 26′ N 73°16′ E) during the rice-growing season (July-October, 2020). The growth and experimental conditions were the same as described above in see section “Initial Screening of Rice Genotypes in a Pot Experiment Under Rainout Zone.” The pots were divided into two sets for two-stage water stress imposition, i.e., (1) well-watered-control and water-stressed for 15 days after 30 DAT (2) well-watered-control and water-stressed for 15 days after 60 DAT. Water stress treatment pots were protected from rain during stress periods. The plants’ physiological and morphological responses were measured and also preserved for biochemical analysis at the end of each stress period. At the stage of harvesting, rice yield and yield attributes were calculated from both sets of control and water-stressed plants.

#### Application of Infrared Thermal Imaging for Measuring Plant Temperature

Infrared (IR) thermal images were taken with a FLIR E6 camera (FLIR Systems Inc., North Billerica, MA, United States). Plants were studied for thermal imaging before and after stress imposition, i.e., 5, 10, and 15 days after stress (DAT) at both early and later growth stages. The FLIR E6 camera was with IR emissivity ranging from0.1 to 0.95, the temperature ranged from −20 to 250°C, spectral ranged 7.5–13 μm, resolution 19,200 pixels (160 × 120), auto hot/cold detection modes, and < 0.06°C thermal sensitivity. The images were taken from a distance of 1.5 m from the plants and simultaneously, visual color images were also saved automatically. A styrofoam sheet was used to minimize the plant background temperature. IR thermal images were analyzed using IR 4.1 FLIR research and development software (FLIR Systems Inc.). The images were taken at 11:00 am, which is the period of high photosynthesis efficiency at Faisalabad (11° 26′ N 73°16′ E). Three-four thermal images from the plant were taken from each of the genotypes per replicate and temperature was averaged.

#### Plant Morphological Responses to Water Deficit Treatments

After the onset of water-stress for 15 days during the early and later growth stages, the plants were sampled for morphological analysis. In this experiment, the root-shoot lengths and the plant fresh and dry weights were measured as described above in see section “Morphological Parameters.”

#### Plant Physio-Biochemical Responses to Water Stress

Different physiological traits of stressed and non-stressed plants were measured after both stages of water deficit treatment from homogenized leaf samples as described in see section “Physiological Parameters.” Chlorophyll content and proline content analysis were carried out as described above in see sections “Leaf Chlorophyll Content” and “Leaf Proline Content,” respectively.

##### Leaf Relative Water Content Analysis

Leaf relative water content (RWC) was estimated according to Barrs and Weatherley (1962). For this parameter, the youngest fully expanded leaves were removed and weighed immediately for measurement of the fresh weight (FW). Then, the leaf segments were soaked 4–6 h in distilled water to measure the turgid weight (TW), and the dry weight (DW) leaf segments were oven-dried for 24 h at 70°C. Three leaves were included from each replication.

The RWC% was calculated by this formula: RWC = (FW–DW/TW–DW) × 100.

##### Leaf Chlorophyll Content Using Soil and Plant Analyzer Development (SPAD) Meter

Chlorophyll content was measured by using a chlorophyll meter (Model: SPAD 502 plus, Japan). SPAD meter was used to measure the chlorophyll contents in the leaves before and after 5, 10, and 15 days during both stages of water stress. The chlorophyll SPAD meter readings were taken from three random positions of one leaf and three different leaves per plant and three plants were selected per replication (Cai et al., 2020).

##### Membrane Stability Index (MSI)

Membrane stability was determined according to the method proposed by Sairam et al. (1997). The leaf discs (1 g) of0.5 cm size were cut from the fully expanded upper leaf washed with distilled water and placed in a test tube containing 15 mL distilled water and incubated at 24°C for 12 h. The electrolytic conductivity (EC) of the solution was measured (C1). The samples were autoclaved at 120°C for 20 min and the EC of the solution was measured after cooling (C2).

MSI (%) was calculated with the following formula: MSI = [1–(C1/C2)] × 100.

#### Analysis of Enzymatic Antioxidants

After the morphological and physio-biochemical analysis, the leaves of rice genotypes were comparatively studied for the activities of different antioxidant enzymes.

##### Catalase (CAT) Activity

Catalase (CAT) activity was determined according to the method described by Zhang et al. (2007). Leaf tissues (0.1 g) from the homogenized sample were ground with 2 ml (0.1 M) sodium phosphate buffer of pH 6.0. Then the homogenate was centrifuged at 13,000 × *g* for 10 min. The supernatant was collected and stored at −18°C until further use. The supernatant (100 μl) was treated with the reaction mixture containing 200 mL of H_2_O_2_ and 30 mL (0.1 M sodium phosphate buffer) having pH 6.0 at room temperature. Distilled water (1.9 ml) with 100 μl sample and 1 ml substrate were added in a cuvette to determine its absorbance at 240 nm using a spectrophotometer (M350, UV visible double beam, CamSpec, United Kingdom). Enzyme activity was expressed on a fresh weight basis, i.e., units/g f. wt.

##### Peroxidase (POD) Activity

For POD estimation, the suspension was prepared by weighing 0.1 g leaves from the homogenized leaf sample, grinding them with a pestle and mortar with 2 ml (0.1 M) sodium phosphate buffer having pH 6.0. Then centrifuged at 13,000 × *g* for 10 min. The supernatant was collected for further analysis and stored at −18°C. The activity of POD was estimated according to the method described by Chance and Maehly (1955). Sodium phosphate buffer (2.8 ml of 50 mM), 800 μl (40 mM) H_2_O_2_, 200 μl guaiacol (substrate), and 60 ml of distilled water were added to prepare the substrate buffer. Substrate buffer (3 ml) and 100 μl supernatant were placed in a cuvette and absorbance at 470 nm were measured. One unit of POD activity was described by a variation of 0.01-unit min^–1^.

##### Phenylalanine Ammonia-Lyase (PAL) Activity

Phenylalanine ammonia-lyase (PAL) enzyme activity was estimated by the method described by Zucker (1965). Leaf tissues (0.1 g) from the homogenized leaf sample were ground with 1 ml (0.1 M) sodium borate buffer of pH 8.8. The homogenate was centrifuged at 13,000 × *g* for 10 min. The supernatant was collected and stored at −18°C for further analysis. The enzyme extract (62.5 μl) and sodium borate buffer (800 μl) were added to the test tube, along with 700 μl (12 mM) phenylalanine. The test tubes were incubated in a water bath at 40°C for 1 h. A 5N HCL (200 μl) was added to stop the reaction. Then, (0.5 ml) of 1 M *Trans*-cinnamic acid (TCA) was added and light absorbance was estimated at 290 nm.

##### Polyphenol Oxidase (PPO) Activity

For the estimation of PPO, 0.1 g leaves from the homogenized sample were ground in pestle and mortar with (2 ml) 0.1 M sodium phosphate buffer of pH 6.0 and centrifuged at 13,000 × *g* for 10 min. The supernatant was taken and stored at −18°C for further analysis. PPO activity was estimated by the method described by Mayer et al. (1965). In the reaction cuvette (1.0 ml), 0.1 M sodium phosphate buffer and (1.0 ml) 0.01 M L-tyrosine (substrate) in HCl were added with (0.9 mL) distilled water. The sample (100 μl) was used to measure the absorbance at 280 nm using a spectrophotometer. The change in absorbance was measured after every 30 s for 2 min and PPO enzyme activity was expressed in min^–1^g^–1^ of fresh weight.

### Effect of Water Stress on Yield Attributes

Plants of each genotype from both stress treatments and controls were manually harvested at maturity. The harvested plants were measured for different growth and yield parameters, i.e., plant height, number of tillers per plant, paddy weight per plant, and paddy yield per pot.

### Statistical Analysis

Data from pot experiments and hydroponic experiments were statistically analyzed by ANOVA and differences between osmotic stressed and controls were compared by the least significant difference (LSD) at 5% (for pot experiment) and 1% confidence level (for the hydroponic experiment) using the software STATISTIX 10.0 (Tallahassee, FL, United States). Pearson correlation coefficient was used for the assessment of associations between different studied plant traits. For interactive studies, the regression analysis of IR temperature with proline content, SPAD, MSI, and RWC were studied using SPSS software (SPSS Inc., NY, United States).

## Results

### Initial Screening of Rice Genotypes Under Net House Conditions

Depending on the visual symptoms of the leaves after 15 days period of water stress imposition, SES scoring was done for all the tested genotypes, i.e., highly tolerant (HT), tolerant (T), moderately tolerant (MT), moderately susceptible (MS), susceptible (S), and highly susceptible (HS). For the evaluation of water-stressed rice leaves, IR-55419-04 and Super Basmati were used as tolerant and susceptible check varieties, respectively. Highly tolerant genotypes were scored as 0, tolerant genotypes as 1, moderately tolerant genotypes as 3, moderately susceptible as 5, susceptible as 7, and highly susceptible as 9 (Supplementary Table 1). After water stress of 15 days, susceptible rice genotypes showed leaf drying followed by chlorosis to dead seedlings (Supplementary Figures 1A–F). Among 26 genotypes 3 were identified as tolerant, 9 moderately tolerant, 5 moderately susceptible, 6 susceptible, and 3 as highly susceptible (Supplementary Table 1).

### Screening of Osmotic Stress Tolerance Under Hydroponic Conditions

Three genotypes were selected on the basis of their phenotypic response and drought tolerance score observed in their initial screening in pots under net house conditions. These genotypes (NIBGE-DT-02, NIBGE-DT-11, and KSK-133) were further screened under control and PEG-simulated osmotic stress hydroponic conditions (Supplementary Table 3).

#### Morphological Responses of Rice Genotypes to Osmotic Stress

**FIGURE 1:**
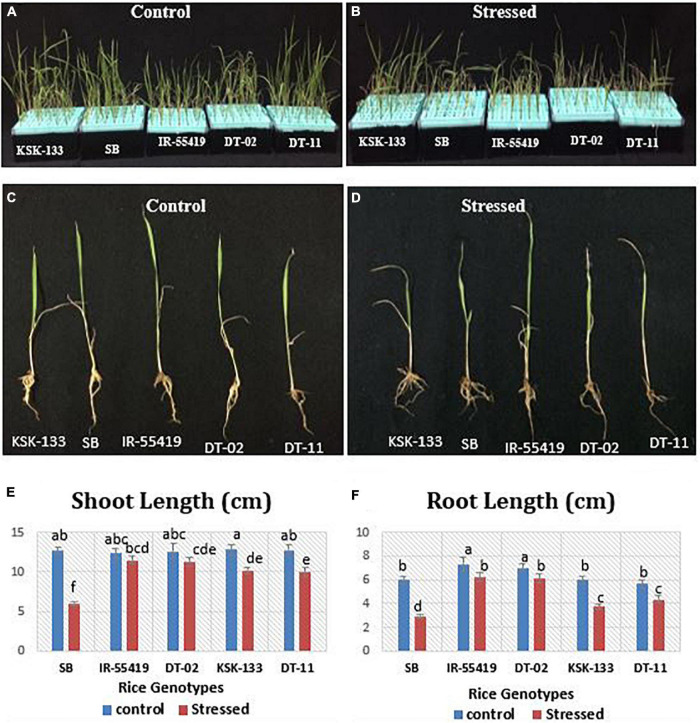
Effect of osmotic stress on rice growth parameters under hydroponic conditions. Rice genotypes: Super Basmati (SB), IR-55419-04, NIBGE-DT-02, KSK-133, and DT-11. **(A)** Control-all tested rice genotypes without osmotic stress. **(B)** Stressed-osmotic stressed [20% Polyethylene Glycol (PEG)] rice seedlings in the hydroponic culture conditions. Morphological response of panel **(C)**. Plants of all tested genotypes under control- (without stress) and panel **(D)**. Plants of all tested genotypes under PEG mediated stressed. Graphical representation of panel **(E)** shoot lengths. **(F)** Root lengths under control-(without stress) and stressed-(20% PEG mediated osmotic stress).

The rice genotypes growing in nutrient solution along with 20% PEG-8000 were studied for shoot and root length (Figures 1A,B). A significant reduction in shoot and root lengths of susceptible genotypes [Super Basmati (SB), NIBGE-DT-11 followed by KSK-133] was observed as compared to tolerant control (IR-55419-04) (Figures 1C,D). The mean values of root length (RL) and shoot length (SL) under osmotic stress showed significant differences among all the tested genotypes (Figures 1E,F). Percent reduction in RL was maximum in SB, i.e., 53.5% followed by KSK-133 (37.2%) and NIBGE-DT-11 (22.9%). At 20%-PEG mediated osmotic stress, the RL ranged from 6.23 to 6.10 cm for IR-55419-04 and NIBGE-DT-02, respectively (Table 1).

**TABLE 1:** Effect of PEG mediated osmotic stress on growth parameters of different rice genotypes under hydroponics.

Control						Stressed				
Rice genotypes	Super Basmati	IR-55419-04	NIBGE-DT-02	KSK-133	NIBGE-DT-11	Super Basmati	IR-55419-04	NIBGE-DT-02	KSK-133	NIBGE-DT-11
**Parameters**										
Shoot length	12.70 ± 0.36^ab^	12.33 ± 0.5^abc^	12.63 ± 0.93^abc^	12.83 ± 0.67^a^	12.77 ± 0.65^ab^	6.00 ± 0.50^f^	11.47 ± 0.64^bcd^	11.30 ± 0.80^cde^	10.17 ± 0.38^de^	10.00 ± 0.56^e^
Root length	6.00 ± 0.30^b^	7.33 ± 0.58^a^	7.03 ± 0.35^a^	6.00 ± 0.30^b^	5.67 ± 0.29^b^	2.79 ± 0.15^d^	6.23 ± 0.40^b^	6.10 ± 0.36^b^	3.77 ± 0.21^c^	4.37 ± 0.32^c^
Shoot fresh weight	0.027 ± 0.003^bcd^	0.031 ± 0.005^ab^	0.032 ± 0.003^a^	0.023 ± 0.003^de^	0.020 ± 0.002^ef^	0.019 ± 0.002^ef^	0.028 ± 0.004^abc^	0.027 ± 0.004^cd^	0.018 ± 0.003^f^	0.016 ± 0.004^f^
Shoot dry weight	0.015 ± 0.002^bcd^	0.019 ± 0.002^ab^	0.020 ± 0.002^a^	0.013 ± 0.001^cde^	0.010 ± 0.002^ef^	0.009 ± 0.001^def^	0.016 ± 0.002^bc^	0.016 ± 0.001^cde^	0.010 ± 0.002^cde^	0.008 ± 0.001^f^
Root fresh weight	0.032 ± 0.003^bc^	0.042 ± 0.004^a^	0.036 ± 0.004^abc^	0.042 ± 0.004^a^	0.033 ± 0.003^bc^	0.023 ± 0.002^de^	0.039 ± 0.003^ab^	0.032 ± 0.003^c^	0.029 ± 0.003^cd^	0.020 ± 0.004^e^
Root dry weight	0.018 ± 0.002^c^	0.031 ± 0.003^a^	0.030 ± 0.002^a^	0.030 ± 0.002^a^	0.019 ± 0.002^c^	0.012 ± 0.001^de^	0.030 ± 0.002^a^	0.024 ± 0.003^b^	0.015 ± 0.002^cd^	0.009 ± 0.001^e^

*Evaluation of osmotic stress on growth parameters: SL-Shoot Length (cm), RL-Root Length (cm), SFW-Shoot Fresh Weight (g), SDW-Shoot Dry Weight (g), RFW-Root Fresh Weight (g), RDW-Root Dry Weight (g), of different rice genotypes: SB (Super Basmati), IR-55419-04, NIBGE-DT-02, KSK-133 and NIBGE-DT-11 under hydroponic conditions. Control- plants grown under sterilized water for 4 days followed by 1/4th Hoagland solution for whole experiment, Stressed-plants grown under sterilized water for 4 days followed by 1/4th Hoagland solution with 5, 10, 15, and 20% PEG mediated osmotic stress, respectively, and duration for each stress level was 4 days. A 28 days old seedlings were removed to measure growth parameters. Data represented as means and means are an average of three biological replicates and there were ten plants per replicate. Means with same letter differ non-significantly at p = 0.01 while different letters show statistical significance according to LSD.*

Osmotic stress treatment significantly reduced shoot fresh weight (SFW) and root fresh weight (RFW) in all the tested genotypes except NIBGE-DT-02 as compared to the tolerant control. SFW at the maximum level of stress ranged from 0.016 to 0.019 g for SB and NIBGE-DT-11, respectively (Table 1). IR-55419-04 showed a minimum percent reduction in the RFW (7.1%) as compared to NIBGE-DT-11 (39.4%). The least reduction in shoot dry weight (SDW) was observed in IR-55419-04 (15.8%) and NIBGE-DT-02 (20%) while KSK-133 and SB showed higher reduction, i.e., 40 and 23.1%, respectively. A similar pattern was observed for root dry weight (RDW) (Table 1).

#### Physiological Responses of Rice Genotypes to Osmotic Stress

**FIGURE 2:**
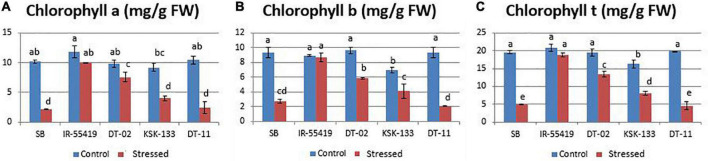
Effect of PEG-induced osmotic stress under hydroponics on chlorophyll a, b, and t of rice genotypes. Genotypes: SB, IR-55419, DT-02, DT-11, and KSK-133. **(A)** Chlorophyll-a content of control-(non-stressed) and stressed-20% PEG induced osmotically stressed plants. **(B)** Chlorophyll-b content of control-(non-stressed) and stressed-20% (PEG) induced osmotically stressed plants. **(C)** Chlorophyll-t (total chlorophyll) of control-(non-stressed) and stressed-20% Polyethylene glycol (PEG) induced osmotic stressed plants in mg/g FW (fresh weight). Ten leaves per plant were homogenized to make a composite sample and three plants per replicate were used. Data represented as mean and means are an average of three biological replicates. According to the least significant difference, means followed by the same letter differ non-significantly at *p* = 0.01 while different letters show statistical significance of genotypes under control and stressed conditions.

Chlorophyll a, b, and total chlorophyll contents were reduced variably in all genotypes under stress conditions as compared to their respective well-watered controls (Figures 2A–C). Higher chlorophyll content with less percent reduction was observed in tolerant check variety (IR-55419-04) followed by NIBGE-DT-02 under osmotic stress. A significant percent decrease in chlorophyll was observed in SB (78.7%) followed by NIBE-DT-11 (76.9%) (Table 2).

**TABLE 2:** Influence of PEG mediated osmotic stress on chlorophyll and proline contents of different rice genotypes under hydroponic conditions.

	Control				Stressed				% decrease in CHL a	% decrease in CHL b	% decrease in CHL t	% increase in Proline
Rice genotypes	CHL a*	CHL b*	CHL t*	Proline**	CHL a*	CHL b*	CHL t*	Proline**				
SB	10.15 ± 0.24^ab^	9.29 ± 0.69^a^	19.56 ± 0.45^a^	16 ± 0.50^c^	2.16 ± 0.08^d^	2.72 ± 0.23^cd^	4.91 ± 0.15^e^	18 ± 1.07^b^	78.70%	70.70%	74.90%	11.11%
IR-55419	11.78 ± 1.01^a^	8.90 ± 0.11^a^	20.83 ± 0.92^a^	17 ± 1.48^b^	10.00 ± 0.05^ab^	8.69 ± 0.60^a^	18.82 ± 0.65^a^	22 ± 2.56^a^	15.10%	2.30%	9.60%	22.70%
DT-02	9.78 ± 0.61^ab^	9.61 ± 0.43^a^	19.50 ± 1.04^a^	16 ± 0.68^b^	7.59 ± 0.83^c^	5.82 ± 0.08^b^	13.51 ± 0.77^c^	20 ± 1.05^a^	22.40%	39.40%	30.70%	20%
KSK-133	9.20 ± 0.73^bc^	6.95 ± 0.35^b^	16.26 ± 1.09^b^	14 ± 0.88^c^	4.02 ± 0.38^d^	4.08 ± 0.98^c^	8.15 ± 0.59^d^	16 ± 1.74^b^	56.30%	41.30%	49.90%	12.50%
DT-11	10.46 ± 0.68^ab^	9.29 ± 0.69^a^	19.88 ± 0.01^a^	15 ± 0.77^c^	2.42 ± 0.99^d^	2.13 ± 0.04^d^	4.58 ± 1.04^e^	17 ± 2.20^b^	76.90%	77.10%	76.90%	11.80%

Effect of osmotic stress on physiological parameters: CHL a-Chlorophyll a, CHL b- Chlorophyll b, CHL t-Chlorophyll t, *- mg/g FW, **- μM/g. f. wt., of different rice genotypes: SB (Super Basmati), IR-55419, DT-02, KSK-133, and DT-11 under hydroponic conditions. Control- plants grown under sterilized water for 4 days followed by 1/4th Hoagland solution for the whole experiment, Stressed-plants grown under sterilized water for 4 days followed by 1/4th Hoagland solution with 5, 10, 15, and 20% PEG mediated osmotic stress, respectively, and duration for each stress level was 4 days. A 28 days old seedlings were removed to measure chlorophyll and proline contents. Data represented as means and means are an average of three biological replicates and there were ten plants per replicate. Means with the same letter differ non-significantly at p = 0.01 while different letters show statistical significance according to LSD.

An increase in proline content (11.11–22.7%) under osmotic stress showed significant genotypic variability. A significant increase in proline concentration was observed in NIBGE-DT-02 (20 μmolg^–1^ fresh weight). While least percent increase in proline accumulation was observed in NIBGE-DT-11 (11.8%) as compared to the tolerant control (Table 2).

### Validation of Rice Genotypes in Pots Under Rain Out Zone

#### Thermal Imaging of Rice Genotypes Response to Water Stress

Results of infrared thermal imaging detected small differences in whole plant temperature of rice genotypes under water stress conditions (Figures 4A–F). During the early and later stages, the plant temperature was almost similar for all the genotypes at 0 days after stress (DAS). At 0DAS, the average temperature during early-stage stress was about 31.9 ± 0.73^°^C and the later stage was 30.9 ± 0.66^°^C, respectively. From 0DAS to 15DAS, in response to water stress, the plant temperature changed gradually in all genotypes during both stages of water withholding. The effect of water stress at 15DAS was significant in SB with higher values of IR temperature (14.7%) during the later growth stage (Table 3).

**TABLE 3:** Infrared thermal imaging to study the effect of water stress on plant temperature during early and later growth stages under net house conditions.

	IR temperature before stress (°c)	IR temperature 5DAS (°c)	% increase in IR-temperature at 5 DAS	IR temperature 10DAS (°c)	% increase in IR-temperature at 10 DAS	IR temperature 15DAS (°c)	% increase in IR-temperature at 15 DAS
**Water stress at 30 DAT**							
Super Basmati	31.8 ± 0.87^a^	34.0 ± 0.94^a^	6.50%	35.7 ± 0.52^a^	10.90%	37.3 ± 0.47^a^	14.70%
DT-02	31.9 ± 0.73^a^	33.0 ± 0.85^b^	3.30%	33.9 ± 0.35^b^	5.90%	34.7 ± 0.61^b^	8.10%
IR-55419	31.6 ± 0.57^a^	32.9 ± 0.40^b^	3.90%	32.9 ± 0.71^b^	3.90%	34.2 ± 0.82^b^	7.60%
**Water stress at 60 DAT**							
Super Basmati	30.4 ± 0.42^a^	32.9 ± 0.73^a^	7.60%	34.1 ± 0.94^a^	10.60%	35.0 ± 0.64^a^	13.10%
DT-02	30.8 ± 0.54^a^	31.9 ± 0.47^b^	3.40%	32.4 ± 0.78^b^	4.90%	33.3 ± 0.59^b^	7.50%
IR-55419	30.9 ± 0.66^a^	31.1 ± 0.54^c^	0.64%	32.0 ± 0.61^b^	3.40%	33.0 ± 0.47^b^	6.40%

*Effect of water withholding for 15 days at 30 days after transplantation (DAT) and 60 DAT on IR (Infrared temperature) of rice plants of different genotypes. Before stress-no stress imposed, 5DAS-(5 days after stress), 10DAS-(10 days after stress), and 15DAS-(15 days after stress). Data represented as means and means are an average of three biological replicates and there were four images per replicate. Means with the same letter differ non-significantly at p = 0.01 while different letters show statistical significance according to LSD.*

#### Morphological Responses of Rice Genotypes to Water Stress

**FIGURE 3:**
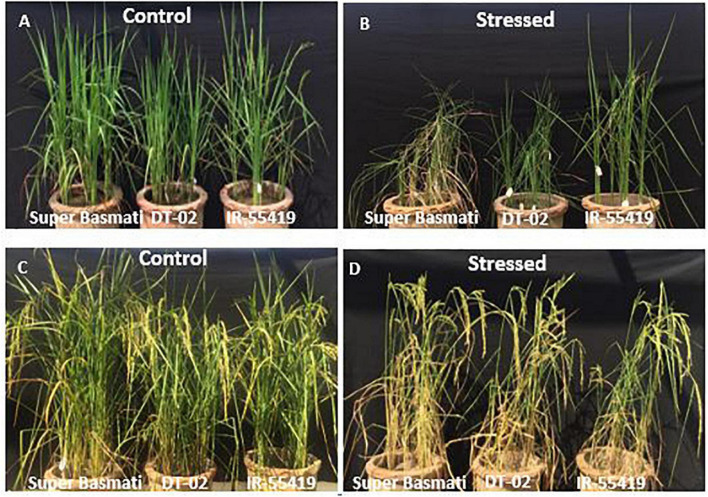
Pictorial view of water stress treatments to rice genotypes during early and later growth stages under net house conditions. Genotypes: SB, IR-55419, and DT-02. **(A)** Control-well irrigated rice plants till 30 days after transplantation (DAS). **(B)** Stressed-water stress rice plants (water withholding for 15 days after 30 DAS) under net house culture conditions. **(C)** Control-well irrigated rice plants till 60 days after transplantation (DAS). **(D)** Stressed-water stress rice plants (water withholding for 15 days after 60 DAS) under net house conditions.

The relative reduction was observed in the growth parameters of rice genotypes under water-stressed conditions, i.e., for 15 days at 30 DAT (Figures 3A,B) and second at 60 DAT (Figures 3C,D) as compared to non-stressed plants. The least and maximum relative reduction was observed in the shoot and root lengths of positive control IR-55419-04 and SB, respectively, under water scarcity. Plant FW was drastically reduced in SB at 15DAS during both stages of water stress. The least reduction in fresh and dry weights under water scarcity was observed in IR-55419-04 and NIBGE-DT-02 (Figures 5A–F).

**FIGURE 4:**
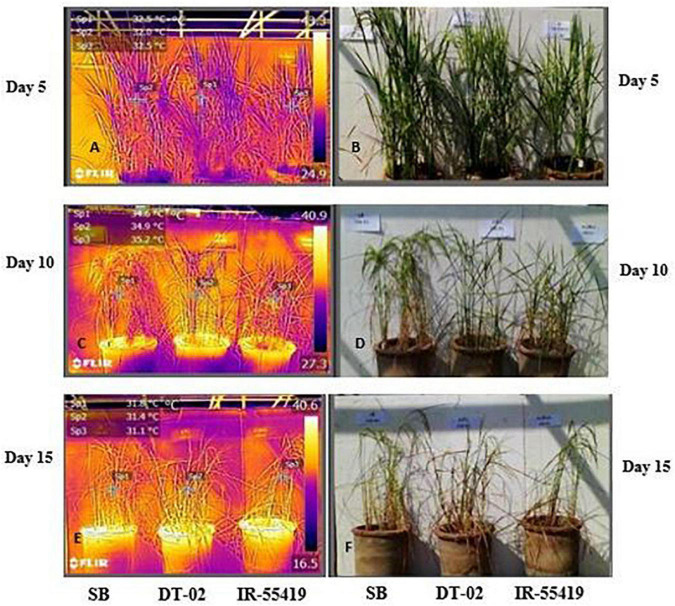
Visual and infrared thermal images of rice genotypes taken by a FLIR T-E6 camera in a net house pot experiment. Genotypes: SB, NIBGE-DT-02, IR-55419-04. **(B,D,F)** Visual images of plants taken at day 5, 10, and 15 after water stress. **(A,C,E)** Thermal images of plants taken at day 5, 10, and 15 after water stress. IR-thermal images were analyzed using IR 4.1 FLIR research and development software (FLIR Systems Inc.).

**FIGURE 5:**
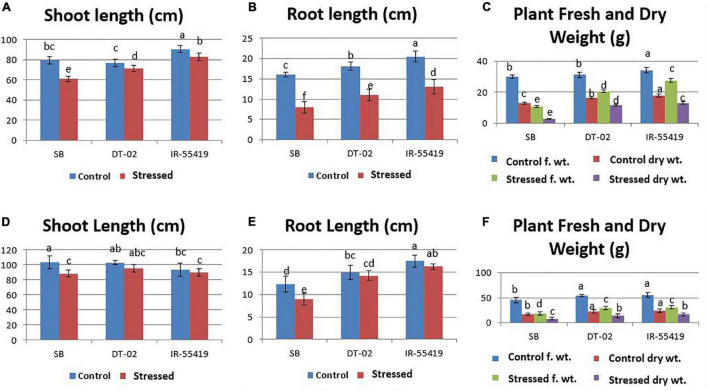
Effect of water stress on rice growth parameters under net house conditions. **(A)** Shoot length (cm). **(B)** Root length (cm). **(C)** Plant fresh and dry weight under control-fully irrigated and stressed-water with holding for 15 days at 30 days after transplantation (DAT). **(D)** Shoot length (cm). **(E)** Root length (cm). **(F)** Plant fresh and dry weight under control-fully irrigated and stressed-water with holding for 15 days at 60 DAT. Rice genotypes: SB, IR-55419, and DT-02. Bars indicate the SD of three biological replicates and each replicate has three plants. Data represented as means and means followed by the same letter differ non-significantly and with different letters show statistical significance at *p* = 0.05 according to LSD.

#### Physio-Biochemical Responses of Rice Genotypes to Water Stress

##### Relative Water Content and Membrane Stability Index (MSI)

The percent RWC of the leaves was measured in the control and stressed plants to understand the effect of water stress on experimental rice genotypes. RWC% was found to be decreased dramatically under drought stress (Table 4A). In the control plants, RWC% was found almost in a similar pattern in all genotypes while water stress resulted in a progressive decline in RWC% values. Under the stress conditions, the highest RWC% during both stages of water scarcity were observed in the genotype IR-55419, followed by DT-02. A maximum percent decrease (45.5 and 43.7%) in RWC was found in genotype SB during water stress at 30 DAT and 60 DAT, respectively (Table 4A).

**TABLE 4A:** Effect of water stress on relative water content (RWC), membrane stability index (MSI) and proline content of rice genotypes during early and later growth stages under net house conditions.

	Control			Stressed			% decrease in MSI	% decrease in RWC	% increase in Proline
	MSI	RWC	Proline*	MSI	RWC	Proline*			
**Water stress at 30 DAT**									
Super Basmati	38.65 ± 3.76^a^	68.08 ± 1.22^b^	23.62 ± 0.98^d^	11.50 ± 3.57^d^	37.09 ± 2.56^c^	29.48 ± 1.07^c^	70.20%	45.50%	19.87%
DT-0-2	22.72 ± 3.42^b^	52.30 ± 5.07^ab^	29.52 ± 1.20^c^	14.67 ± 1.54^cd^	45.07 ± 4.54^b^	46.72 ± 2.3^a^	35.40%	13.80%	36.80%
IR-55419	17.61 ± 1.51^a^	60.40 ± 5.49^a^	27.00 ± 1.01^d^	16.83 ± 1.19^a^	55.79 ± 1.04^a^	40.09 ± 1.59^b^	21.50%	7.60%	32.65%
**Water stress at 60 DAT**									
Super Basmati	15.27 ± 1.49^a^	55.73 ± 3.68^a^	29.89 ± 1.51^c^	8.71 ± 0.34^c^	31.39 ± 3.99^c^	46.00 ± 1.39^b^	42.90%	43.70%	35.02%
DT-02	12.29 ± 0.95^b^	78.32 ± 0.76^a^	30.43 ± 1.06^c^	11.18 ± 0.53^bc^	73.99 ± 1.43^ab^	56.25 ± 1.40^a^	9%	5.50%	45.90%
IR-55419	19.09 ± 2.16^bc^	73.29 ± 1.51^ab^	17.52 ± 0.49^e^	16.40 ± 0.47^bcd^	70.84 ± 4.98^b^	30.07 ± 1.41^c^	14.10%	3.30%	41.70%

Evaluation of water stress on MSI-Membrane stability index, RWC-Relative Water Content, and proline *- μM/g. f. wt., of different rice genotypes: Super Basmati, IR-55419, and DT-02 under net house conditions. Control- well-irrigated plants, Stressed-water stress for 15 days at 30 DAT, and water stress for 15 days at 60 DAT. After each water stress plants were removed to measure MSI, RWC, and proline content. Data represented as means and means are an average of three biological replicates and each replicate has three plants (ten leaves per plant). Data represented as means and means with the same letter differ non-significantly and with different letters differ significantly at p = 0.05 according to LSD.

Increased electrolyte leakage (EL) under water scarcity points toward the increase in the plant cell membrane injury. Water stress caused a decrease in MSI during the period of stress and the magnitude of the decline in MSI for susceptible rice genotype (SB-70.20%, 42.90%) was greater than that of the tolerant genotype NIBGE-DT-02 (35.90%, 9%) during water stress at 30 DAT and 60 DAT, respectively (Table 4A).

##### Proline and Chlorophyll Content

Under water stress proline concentrations (μ molg^–1^ FW) of rice, genotypes were increased to a variable extent as compared to their respective controls during both stages of stress treatments. The water stress-tolerant genotype NIBGE-DT-02 accumulated the highest concentration (46.72, 56.25 μ molg^–1^ FW) of proline at 15DAS during water stress at 30 DAT and 60 DAT, respectively. SB showed the least percent increase (19.87, 35.02%) in proline content in both stages of water stress (Table 4A).

Rice genotypes for chlorophyll a, b, and total chlorophyll differed variably under water scarcity. Higher values of chlorophyll contents after positive control were observed in NIBGE-DT-02 during the early and later stages of water stress. SB showed the highest percent reduction in total chlorophyll content (39.14, 34.50%) in water stress at 30 DAT and 60 DAT, respectively (Table 4B).

**TABLE 4B:** Effect of water stress on chlorophyll content and SPAD values in different rice genotypes during early and later growth stages in a pot experiment under net house conditions.

	Control				Stressed				% decrease in CHL a	% decrease in CHL b	% decrease in CHL t	% decrease in SPAD values
	CHL a*	CHL b*	CHL t*	SPAD values	CHL a*	CHL b*	CHL t*	SPAD values				
**Water stress** **at 30 DAT**												
Super Basmati	26.06 ± 0.55^a^	15.13 ± 0.73^a^	41.52 ± 1.28^a^	81.97 ± 0.55^a^	15.02 ± 0.72^e^	10.07 ± 0.21^d^	25.27 ± 0.52^d^	71.17 ± 0.86^c^	42.40%	33.40%	39.14%	13.80%
DT-02	23.20 ± 0.83^b^	13.75 ± 0.42^bc^	37.25 ± 1.26^b^	81.82 ± 0.74^a^	18.76 ± 0.90^d^	12.58 ± 0.31^c^	31.57 ± 1.22^c^	77.60 ± 0.52^b^	19.13%	8.50%	15.20%	5.20%
IR-55419	23.36 ± 0.69^b^	13.97 ± 0.30^ab^	37.63 ± 0.60^b^	82.02 ± 0.94^a^	20.80 ± 0.69^c^	12.92 ± 0.63^bc^	33.99 ± 1.63^c^	79.10 ± 0.58^a^	10.90%	7.50%	9.70%	3.60%
**Water stress** **at 60 DAT**												
Super Basmati	32.79 ± 0.56^ab^	23.22 ± 0.14^c^	56.42 ± 0.71^c^	81.79 ± 0.41^a^	21.04 ± 0.38^e^	15.67 ± 0.45^e^	36.97 ± 0.83^f^	74.15 ± 0.48^c^	35.80%	32.50%	34.50%	9.30%
DT-02	31.37 ± 0.85^c^	18.53 ± 0.11^d^	50.30 ± 0.97^d^	81.83 ± 0.14^a^	28.12 ± 0.38^d^	17.68 ± 0.48^d^	46.15 ± 0.86^e^	77.73 ± 0.35^b^	10.40%	3.90%	8.30%	5%
IR-55419	33.39 ± 0.22^a^	29.33 ± 0.54^a^	63.11 ± 0.76^a^	81.79 ± 0.24^a^	31.89 ± 0.50^bc^	27.36 ± 0.65^b^	59.63 ± 0.14^b^	79.89 ± 0.47^a^	4.50%	6.70%	5.50%	2.30%

Evaluation of water stress on CHL a-Chlorophyll a, CHL b- Chlorophyll b, CHL t-Chlorophyll t, *-mg/g FW, SPAD- Soil and Plant Analyzer Development, of different rice genotypes: Super Basmati, IR-55419, and DT-02 under net house conditions. Control- well-irrigated plants, Stressed-water stress for 15 days at 30 DAT, and water stress for 15 days at 60 DAT. After each water stress plants were removed to measure MSI, RWC, and proline content. Data represented as means and means are an average of three biological replicates and each replicate has three plants (ten leaves per plant). Data represented as means and means with the same letter differ non-significantly and with different letters differ significantly at p = 0.05 according to LSD.

The SPAD values from the leaves of rice genotypes showed a similar pattern with the chlorophyll contents of respective genotypes. The least percent reduction in SPAD values was observed for NIBGE-DT-02 (5%) later stage (60 DAT) water stress (Table 4B).

##### Enzymatic Antioxidants

**FIGURE 6:**
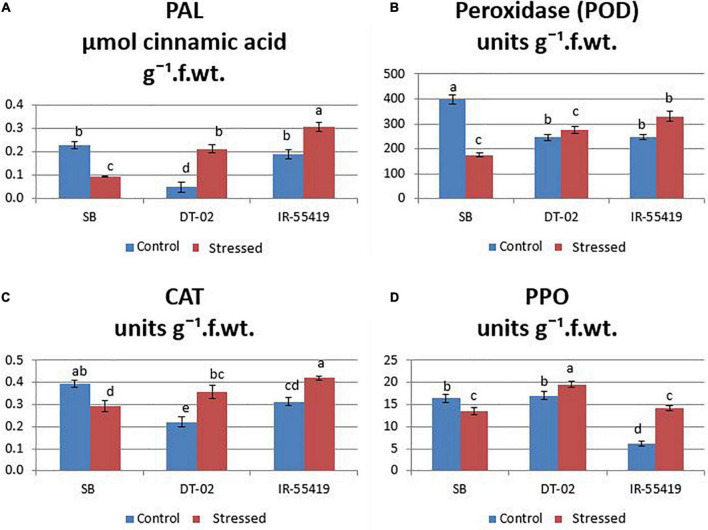
Induction of defense-related enzymes. **(A)** Phenylalanine ammonia lyase (PAL). **(B)** Peroxidase (POD). **(C)** Catalase (CAT). **(D)** Polyphenol oxidase (PPO) in rice plants of genotypes SB, IR-55419 and DT-02 under control-fully irrigated and stressed-water with holding for 15 days at 30 days after transplantation (DAT). Bars indicate the SD of three biological replicates and each replicate has three plants (ten leaves per plant). Data represented as means and means with the same letter differ non-significantly at *p* = 0.05 according to LSD.

**FIGURE 7:**
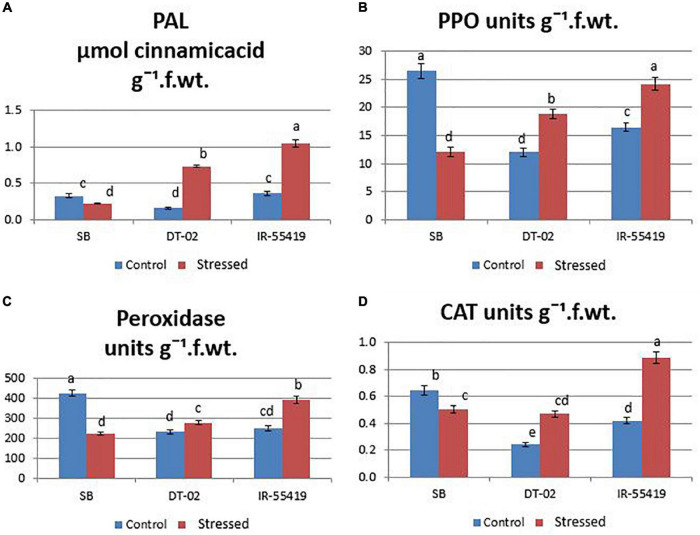
Induction of defense-related enzymes. **(A)** Phenylalanine ammonia-lyase (PAL). **(B)** Polyphenol oxidase (PPO). **(C)** Peroxidase (POD). **(D)** Catalase (CAT) in rice plants of genotypes SB, IR-55419, and DT-02 under control-fully irrigated and stressed-water withholding for 15 days at 60 days after transplantation (DAT). Bars indicate the SD of three biological replicates and each replicate has three plants (ten leaves per plant). Data represented as means and means with the same letter differ non-significantly at *p* = 0.05 according to LSD.

The results showed that the concentration of antioxidant enzyme was found maximum in the tolerant and minimum in susceptible rice genotypes under water scarcity. However, the accumulation of enzymes varied between all the tested genotypes. Under water stress, the highest accumulation of antioxidants (CAT:0.36, POD:276.03, PAL:0.21, and PPO: 19.57 units g^–1^ f. wt.) during water stress at 30 DAT (Figures 6A–D) and (CAT:0.47, POD:280, PAL:0.73, and PPO:18.86 units g^–1^ f. wt.) at 60 DAT water stress (Figures 7A–D) were recorded in NIBGE-DT-02 followed by the positive control (IR-55419-04). The susceptible genotype (SB) under stress showed less accumulation of defense-related enzymes as compared to non-stressed conditions.

#### Effect of Water Stress on Rice Yield

Irrespective of rice genotype, water stress reduced the plant height. A high reduction in plant height was recorded in SB while less reduction was observed in IR-55419 followed by DT-02 under water stress conditions as compared to their respective controls in both stages of stress. Drought stress reduced the number of tillers per plant in all tested rice genotypes. Maximum reduction in tiller number was recorded in drought susceptible genotype SB. Drought stress significantly reduced the grain yield in SB (82.30%) during water stress at 30 DAT while the least reduction in NIBGE-DT-02 (35.90%) during water stress at 60 DAT (Table 5). Percent decrease in plant height in all genotypes was more during early-stage water stress as compared to a later stage, while percent reduction in plant fresh weight was more during early-stage water scarcity because of low tillering so in correspondence to this percent decrease in grain yield was more during water stress at 30 DAT (Table 5).

**TABLE 5:** Effect of water stress on yield attributes of rice genotypes during early and later growth stages in a pot experiment under net house conditions.

	Control				Stressed				% decrease in plant height	% decrease in plant weight	% decrease in grain weight
	Plant Height*	No. of tillers per plant	Plant weight**	Grain weight***	Plant Height*	No. of tillers per plant	Plant weight**	Grain weight***			
**Water stress** **at 30 DAT**											
Super Basmati	111.50 ± 3.70^a^	9.8 ± 2.5^ab^	70.10 ± 2.46^a^	27.95 ± 2.1^a^	81.50 ± 1.29^e^	3.8 ± 0.5^c^	18.85 ± 0.91^f^	4.95 ± 3.0^e^	26.90%	73.11%	82.30%
DT-02	91.75 ± 1.71^c^	9.8 ± 1.5^ab^	42.90 ± 2.03^b^	34.05 ± 1.34^a^	73.75 ± 3.30^f^	5.8 ± 2.8^c^	20.00 ± 1.19^ef^	11.85 ± 0.92^cd^	19.60%	53.40%	65.20%
IR-55419	87.25 ± 2.99^d^	10.5 ± 2.4^a^	43.68 ± 2.10^b^	31.25 ± 3.46^a^	72.50 ± 2.08^f^	5.3 ± 1.3^c^	25.68 ± 1.33^c^	16.6 ± 1.13^bc^	16.90%	41.20%	46.88%
**Water stress** **at 60 DAT**											
Super Basmati	100.50 ± 4.80^a^	12 ± 1.83^a^	58.58 ± 2.77^a^	29.85 ± 4.6^a^	87.75 ± 1.71^b^	6 ± 0.82^b^	29.50 ± 1.29^e^	6.55 ± 0.92^e^	12.60%	49.60%	78.06%
DT-02	97.50 ± 4.20^a^	11.8 ± 2.06^a^	61.13 ± 2.60^a^	16.95 ± 1.1^bc^	88.00 ± 2.16^b^	6.5 ± 0.58^b^	35.23 ± 1.50^cd^	10.85 ± 0.5^d^	9.70%	42.40%	35.90%
IR-55419	79.25 ± 1.71^c^	10.5 ± 1.91^a^	58.65 ± 3.00^a^	20.2 ± 1.8^b^	75.00 ± 1.83^d^	7.3 ± 0.50^b^	37.88 ± 1.62^bc^	15.1 ± 1.3^cd^	5.40%	35.40%	25.20%

Evaluation of water stress on yield attributes: *plant height (cm), no. of tillers, **plant weight (g) and ***grain weight (g), of rice genotypes: Super Basmati, IR-55419, and DT-02 under net house conditions. Control- well-irrigated plants, Stressed-water stress for 15 days at 30 DAT, and water stress for 15 days at 60 DAT. After each water stress plants were removed to measure MSI, RWC, and proline content. Data represented as means and means are an average of three biological replicates and each replicate has three plants. Data represented as means and means with the same letter differ non-significantly and with different letters differ significantly at p = 0.05 according to LSD.

#### Correlation Analysis Among Morpho-Physiological and Biochemical Traits

**FIGURE 8:**
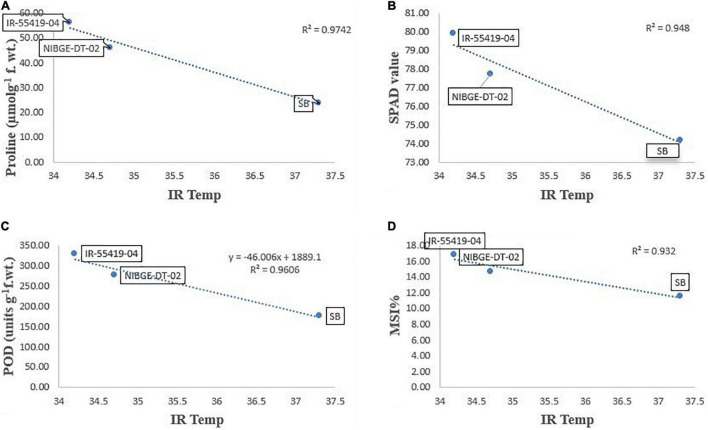
Correlation analysis of Infrared (IR) temperature with different traits for three tested genotypes under early-stage stress conditions. **(A)** Relationship between IR-temperature and proline content. **(B)** Relationship between IR-temperature and SPAD values. **(C)** Relationship between IR-temperature and POD enzyme. **(D)** Relationship between IR-temperature and MSI%. Relationships were studied by correlation analysis using SPSS software. Means are an average of four biological replicates at *p* = 0.05 according to the LSD.

**FIGURE 9:**
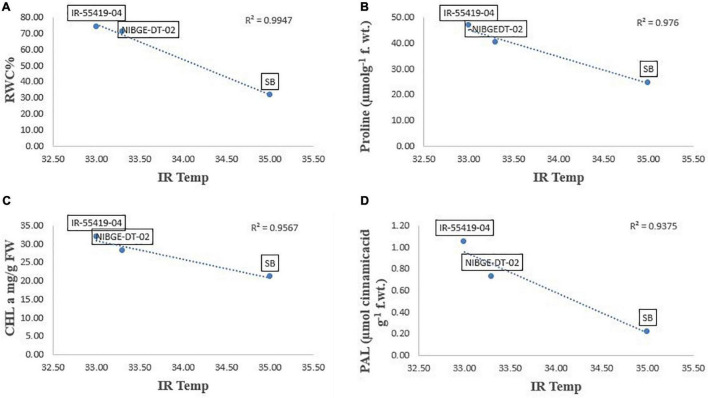
Correlation analysis of Infrared (IR) temperature with different traits for three tested genotypes under later-stage stress conditions. **(A)** Relationship between IR-temperature and RWC%. **(B)** Relationship between IR-temperature and proline content. **(C)** Relationship between IR-temperature and chlorophyll a (CHL a). **(D)** Relationship between IR-temperature and PAL enzymes. Relationships were studied by correlation analysis using SPSS software. Means are an average of four biological replicates at *p* = 0.05 according to the LSD.

Pearson’s correlation analysis was performed on different morphological and physio-biochemical traits of water-stressed plants (studied during early and later growth stage water stress). The results indicated that (at *p* < 0.05) positive correlation among all the tested genotypes with different traits was expressed in bold form while the negative correlation with a negative sign. So, on this basis, a significant correlation with multiple traits related to stress tolerance was easily identified during both stage stress conditions (Tables 6A,B). A negative correlation was observed between infrared (IR) temperature with (proline content, SPAD values, MSI and POD) during early-stage and with (RWC, proline content, MSI and PAL) during later-stage water stress (Figures 8, 9A–D).

**TABLE 6A:** Correlation matrix for morphological and physio-biochemical traits during early-stage water stress in a pot experiment under net house conditions.

Traits	PPO	SL	RWC	PAL	CAT	PDW	CHL b	POD	CHL a	MSI	RL	PFW	CHL t	Proline
PPO	1													
SL	0.08	1												
RWC	0.02	1	1											
PAL	0.15	1	**0.99**	1										
CAT	0.15	1	**0.99**	1	1									
PDW	0.47	**0.92**	**0.89**	**0.94**	**0.94**	1								
CHL b	0.49	**0.91**	**0.88**	**0.94**	**0.93**	1	1							
POD	0.27	**0.98**	**0.97**	**0.99**	**0.99**	**0.98**	**0.97**	1						
CHL a	0.27	**0.98**	**0.97**	**0.99**	**0.99**	**0.98**	**0.97**	1	1					
MSI	0.21	**0.99**	**0.98**	1	1	**0.96**	**0.95**	1	1	1				
RL	0.21	**0.99**	**0.98**	1	1	**0.96**	**0.96**	1	1	1	1			
PFW	0.19	**0.99**	**0.98**	1	1	**0.96**	**0.95**	1	1	1	1	1		
CHL t	0.35	**0.96**	**0.94**	**0.98**	**0.98**	**0.99**	**0.99**	1	1	**0.99**	**0.99**	**0.99**	1	
Proline	0.31	**0.97**	**0.96**	**0.99**	**0.99**	**0.98**	**0.98**	1	1	**0.99**	1	**0.99**	1	1

*Relationship of water-stressed- 15 days of water stress at 30 DAT, morphological and physio-biochemical traits: SL-Shoot length, RL-Root length, RWC-Relative water content, PFW-plant fresh weight, PDW-plant dry weight, CAT-catalase, POD-peroxidase, PPO- Polyphenol oxidase, PAL-Phenylalanine ammonia-lyase, CHL a-chlorophyll a, CHL b-chlorophyll b, CHL t-total chlorophyll and MSI-membrane stability index. Values in bold differ from 0 with level of significance p = 0.05.*

**TABLE 6B:** Correlation matrix for morphological and physio-biochemical traits during later-stage water stress in a pot experiment under net house conditions.

Traits	Proline	PAL	CAT	POD	PPO	SL	MSI	CHL b	CHL t	PFW	PDW	CHL a	RL	RWC
Proline	1													
PAL	**0.99**	1												
CAT	0.69	**0.75**	1											
POD	0.53	0.51	**0.78**	1										
PPO	0.61	0.59	**0.77**	**0.97**	1									
SL	–0.29	–0.43	–0.43	0.22	0.17	1								
MSI	–0.03	–0.19	–0.53	0	–0.01	**0.86**	1							
CHL b	0.37	0.38	0.43	0.45	0.52	–0.09	–0.2	1						
CHL t	0.36	0.32	0.3	0.5	0.55	0.21	0.12	**0.95**	1					
PFW	–0.27	–0.37	–0.44	0	0.01	0.67	0.58	0.49	0.68	1				
PDW	–0.04	–0.13	–0.33	–0.01	0.03	0.48	0.5	0.63	0.79	**0.95**	1			
CHL a	0.3	0.21	0.1	0.49	0.52	0.53	0.48	0.76	**0.93**	**0.81**	**0.86**	1		
RL	0.48	0.41	0.01	0.13	0.22	0.14	0.34	0.74	**0.83**	0.65	**0.84**	**0.83**	1	
RWC	0.5	0.41	–0.07	0.12	0.21	0.26	0.51	0.59	**0.74**	0.64	**0.81**	**0.82**	**0.97**	1

*Relationship of water-stressed- 15 days of water stress at 60 DAT, morphological and physio-biochemical traits: SL-Shoot length, RL-Root length, RWC-Relative water content, PFW-plant fresh weight, PDW-plant dry weight, CAT-catalase, POD-peroxidase, PPO- Polyphenol oxidase, PAL-Phenylalanine ammonia-lyase, CHL a-chlorophyll a, CHL b-chlorophyll b, CHL t-total chlorophyll and MSI-membrane stability index. Values in bold differ from 0 with level of significance p = 0.05.*

## Discussion

In the scenario of climatic shift, water scarcity is one of the most common abiotic stresses that hinders rice growth through alteration in many morphological, physiological, and biochemical responses (Chaudhry and Sidhu, 2021). Usually, rice genotypes are being selected on the basis of their tolerance to water scarcity using different drought-related morpho-physiological and biochemical approaches (Panda et al., 2021). The present study effectively combined these approaches with the useful tool of SPAD meter with further validation of drought tolerance in rice genotypes using the technology of infrared thermal imaging. Furthermore, the selected genotype was subjected to water scarcity at early (vegetative) as well as later (reproductive) growth stages to ensure its tolerance throughout the whole crop season.

In our experiment, initially, 28 rice genotypes including one tolerant (IR-55419-04) and one susceptible check (Super Basmati) variety with 26 advance lines were screened for water stress tolerance in pots under net house conditions. Among the tested genotypes, 3 were identified as tolerant, 9 moderately tolerant, 5 moderately susceptible, 6 susceptible, and 3 as highly susceptible to water deficit conditions (Supplementary Table 1). Three promising genotypes (NIBGE-DT-02, NIBGE-DT-11, and KSK-133) were selected based on the standard evaluation system (SES) scoring for drought tolerance (Supplementary Table 3). SES scoring for rice genotypes is a reliable measure of drought tolerance and reflects dehydration in plant tissues (Swapna and Shylaraj, 2017). The results of the present study in pots under net house conditions showed that water withholding for 15 days at 30 days after transplantation significantly affected the plants’ phenotypic response as indicated by leaf drying to dead seedlings (Supplementary Table 2).

Three selected genotypes (NIBGE-DT-02, NIBGE-DT-11, and KSK-133) were subjected to Polyethylene glycol (PEG-20%) mediated osmotic stress under a hydroponic system in a growth room experiment. PEG-induced osmotic stress reduced all the growth parameters of rice genotypes to a variable extent (Nahar et al., 2018). In the present study, a higher level of PEG (20%) stress reduced the growth attributes of all the tested rice genotypes, the maximum reduction in the shoot length (21.7%) of NIBGE-DT-11 and in the root length (37.2%) of KSK-133 was observed (Table 1). Shirazi et al. (2019) reported that the reduction in growth parameters was also reflected in the physiological responses of susceptible rice genotypes, i.e., IR-8 and B-60-B. Genotype NIBGE-DT-02 achieved better growth in hydroponics osmotic stress as well as under variable water withholding stages in pots under net house conditions. NIBGE-DT-02 showed a minimum reduction in the shoot (10.5%) and root (13.2%) lengths under hydroponics (Table 1). Franco et al. (2011) ratified the better root growth of plants under stress conditions as an important index for the selection of drought-tolerant cultivars. In the present study, NIBGE-DT-02 revealed a minimum reduction in root growth concomitant with the least reduction in plant fresh and dry weights under 15-days water withholding at 30 and 60 DAT (Figures 5A–F).

Chlorophyll is an important plant photosynthetic pigment, determining plant growth and development. Under osmotic stress, a significant decrease in chlorophyll a (76.9%) and b (77.1%) were observed in the genotype NIBGE-DT-11. Genotypes NIBGE-DT-02 showed the least reduction in chlorophyll a (22.4%) and chlorophyll t (30.7%) under osmotic stress as compared to the susceptible check variety (Table 2). Nahar et al. (2018) reported that rice variety (SN09) showed significantly minimum chlorophyll a and b content as compared to the tolerant variety (SN03) under PEG (20%) induced stress. Moreover, NIBGE-DT-02 showed a minimum reduction in chlorophyll a (10.4%), b (3.9%), and t (8.30%), while a similar pattern of NIBGE-DT-02 was observed with a minimum (5%) reduction in SPAD values under 15-days water withholding at 60 days after transplantation (Table 4B). According to Nahakpam (2017), the reduction in chlorophyll content might be due to the formation of active oxygen species (AOS) that affects the stability of the chloroplast membrane and causes the chlorophyll degradation under water stress conditions.

Plants produced proline as an important osmolyte to maintain protein conformation and stabilize the membranes at a low level of leaf water potential. Likewise, an increased accumulation of proline was observed in genotype NIBGE-DT-02 (20%) followed by the tolerant check variety (22.7%) as compared to the susceptible check variety (Table 2). In plants, the relative increase of proline content under drought stress has been proposed as a potential indicator for the selection of drought-tolerant varieties (Dien et al., 2019). Abdula et al. (2016) reported that the increased biosynthesis of proline enhanced abiotic stress tolerance in rice genotypes. In this study maximum increase in proline content was observed in genotype NIBGE-DT-02 during early (32.65%) and later (41.7%) growth-stage water stress followed by tolerant check variety (Table 4). As reported by Saha et al. (2019), proline is a key factor involved in the mechanism of tolerance against 10 days of water stress as BRRI-dhan-56 showed 3.7 folds increase in proline accumulation under water withholding at 21 days after sowing.

Relative water content (RWC) is considered as an effective physiological parameter to measure the water content of plants under control and water deficit conditions (Gupta et al., 2020; Zhu et al., 2020). According to Puangbut et al. (2018) the drought-tolerant variety maintained higher RWC as indicated by low reduction (21.6%) in comparison with the susceptible variety (35%). In our present study, tolerant genotype NIBGE-DT-02 exhibited less reduction in RWC (5.5%) with the maximum accumulation of proline osmolyte (45.9%) under water deficit conditions at 60 DAT (Table 4A). Swapna and Shylaraj (2017) stated that the ability to retain water content (74.37%) in rice variety (Neeraja) under drought stress may be due to osmolyte accumulation in cells or due to rigidness of cell wall.

The increase in EL values points toward the increase in the cell membrane injury induced by the water stress (Al-Ashkar et al., 2016). In the present study highest electrolyte leakage (EL) was observed in Super Basmati as indicated by maximum reduction (70.2%) in membrane stability index (MSI) under water stress at 30 days after transplantation. NIBGE-DT-02 (35.4%) followed by tolerant check variety IR-55419-04 (21.5%) exhibited the least reduction in MSI under 15 days water stress at 30 DAT (Table 4A).

In this study, the plants were also studied for the induction of stress-related antioxidant enzymes. The plants of NIBGE-DT-02 showed a significant increase in PAL enzyme activity (0.73 μmol cinnamic acid/g F. wt.) with a concomitant increase in POD, CAT, and PPO (units/g. F. wt.) under 15 days water stress at 60 DAT as compared to its well-watered control (Figure 7). Sahebi et al. (2018) reported that in rice, water scarcity leads to the formation of ROS in various cellular compartments (mitochondria, chloroplast, and peroxisomes). The enhanced activities of ROS scavengers might be one strategy of this genotype for reducing oxidative damage and improving the drought resistance in rice plants (Sachdev et al., 2021).

Sabar and Arif (2014) reported significant variation in rice genotypes for yield-related attributes. Severe water scarcity at vegetative (early) and reproductive (later) growth stages is needed to screen segregating germplasm because stress imposition of different types exposed the genetic variation of genotypes due to the underlying different mechanisms of drought tolerance (Mumtaz et al., 2019). In our experiment, water stress imposed during the early growth stage coincides more or less with the onset of vegetative (before flowering) and later growth stage stress with the reproductive stage. Wang et al. (2017) reported that biomass production is largely affected under vegetative growth stage water scarcity. In the present study, the percent decrease in plant height in all genotypes was more during the early-stage water stress as compared to the later stage, while percent reduction in plant fresh weight was more during early-stage water scarcity because of low tillering so in correspondence to this percent decrease in grain yield was more during water stress at 30 days after transplantation (Table 5).

Correlation analysis between morphological, physiological, and biochemical traits of stressed rice plants indicated that root length was strongly correlated with PAL, relative water content, (chl b), catalase, and plant dry weight while relative water and proline contents strongly correlated with catalase, PAL, and membrane stability index. These results depicted that an increase in root growth had significant effects on plant physiological and biochemical responses to early-stage water stress (Table 6A). Proline and relative water contents have been proposed as key factors involved in the mechanism of tolerance to water stress (Saha et al., 2019). The strong correlation between RWC and RL under water stress during both growth stages (Tables 6A,B) showed that RL played a significant role in plant survival under low water potential and these traits (RWC and proline) could be used as a good marker to determine drought tolerance in rice plants. Furthermore, in the present study regression analysis showed a negative correlation between plant temperature (IRTI) and proline content (*r*^2^ = 0.97) under 15-days water stress condition at 30 days after transplantation while plant temperature under water stress at 60 DAT also had a strong correlation with RWC (*r*^2^ = 0.99). Previously, Carroll et al. (2017) argued that plant temperature increases with decreased plant available water. Though it is normal for plant temperature to rise during the day and reduce throughout the night in relative to well-watered control, the water-stressed plants have less evaporation cooling with lower rates of transpiration and hence have a higher temperature at daytime. Thus, the measurement of plant temperature quantifies the degree of plant water stress if compared to the well-watered control plants. In this study, all the rice genotypes showed a variable decline in RWC (Table 3) and an increase in plant temperature (Table 4A) of water-stressed plants as compared to the non-stressed plants. The rice genotype NIBGE-DT-02 showed less percent decrease (5.5%) in RWC and less percent increase (7.5%) in IR temperature as compared to the susceptible variety under water stress at 60 DAT (Tables 3, 4A). Saleem et al. (2020) reported that the ability of a plant to keep leaf temperature cooler was associated with drought stress tolerance.

The correlation between marker traits (proline and RWC) and plant temperature highlighted the significance of infrared thermal imaging as an effective tool for the indication of drought tolerance in plants. These findings provide a foundation for future research directed to utilize the IRTI approach for the selection of potent rice genotypes better adapted to water scarcity from a wide germplasm collection. Additionally, drought stress affects more or less at every growth stage causing a reduction in yield attributes. The selected genotype NIBGE-DT-02 (drought-tolerant introgression) of the recipient variety Super Basmati (aromatic, long-grain, and sensitive to water stress) was improved significantly and comparatively more tolerant to water stress irrespective of the growth stage as this genotype gave significantly higher yield than the susceptible genotype. Consequently, sustainable rice production under this climatic shift will bring more opportunities for food security and prosperity of the country.

## Conclusion

Drought stress has been increased drastically due to climate change, which limits the growth and yield of rice worldwide. Therefore, the present study aimed for the reliable selection of drought-tolerant genotypes by integrating morpho-physiological and biochemical approaches with *in situ* technology of infrared thermal imaging (IRTI) to sustain crop production under water scarcity. The selected rice genotype NIBGE-DT-02 has significant production of osmoregulator (proline), antioxidants, relative water content, better yield, and tolerance to water stress irrespective of the growth stage. Our study suggests that the correlation between infrared thermal imaging and different physio-biochemical responses provides a foundation for future research directed to utilize the IRTI approach for the selection of potent rice genotypes better adapted to water scarcity from wide germplasm collection. These findings can further be utilized for breeding programs to address the food security issues in this alarming situation of climate change.

## Data Availability Statement

The original contributions presented in the study are included in the article/Supplementary Material, further inquiries can be directed to the corresponding author.

## Author Contributions

NM did the overall execution of the experiment, analytical work, collection of infrared thermal images and software analysis, collection of data after morpho-physiological and biochemical analysis of leaves, organization of resulting data, and writing up and revision of manuscript. SYa and MAr contributed to the planning, designing and finalization of basic idea of experiments and overall supervision during analytical work, revised, and finalized the manuscript. MAs did the arrangement and provision of rice seeds, contributed in study basic idea and planning of net house experiment, and help in data collection. MY and KE performed data analysis and reviewed the manuscript. SYo helped in plant biochemical analysis and data execution. ZS and HS helped in the analysis of plant stress physiological responses. All authors contributed to the article and approved the submitted version.

## Conflict of Interest

The authors declare that the research was conducted in the absence of any commercial or financial relationships that could be construed as a potential conflict of interest.

## Publisher’s Note

All claims expressed in this article are solely those of the authors and do not necessarily represent those of their affiliated organizations, or those of the publisher, the editors and the reviewers. Any product that may be evaluated in this article, or claim that may be made by its manufacturer, is not guaranteed or endorsed by the publisher.

## Supplementary Material

Supplementary Figure 1 Initial screening of rice genotypes under net house conditions. **(A)** Control-well irrigated rice plants till 30 days after transplantation (DAT). **(B)** Stressed-water stress rice plants (water withholding for 15 days after 30 DAT) under net house conditions. **(C)** Control-well irrigated five rice genotypes viz, IR-55419-04, NIBGE-DT-02, SB, NIBGE-DT-11, and KSK-133 selected from initial screening. **(D)** Stressed-water stress five rice genotypes viz, IR-55419-04, NIBGE-DT-02, SB, NIBGE-DT-11, and KSK-133. **(E)** Control-leaves of plants of genotypes IR-55419-04, NIBGE-DT-02, SB, NIBGE-DT-11, and KSK-133, respectively, under well water conditions. **(F)** Stressed- leaves of plants of genotypes IR-55419-04, NIBGE-DT-02, SB, NIBGE-DT-11 and KSK-133, respectively, under water deficit conditions.
