# Supplementary material for: Integrated Analysis of Osmotic Stress and Infrared Thermal Imaging for the Selection of Resilient Rice Under Water Scarcity
Source: Front Plant Sci. 2022 Feb 14;13:834520. doi: 10.3389/fpls.2022.834520 (PMC8882677; doi:10.3389/fpls.2022.834520)
Supplement: Supplementary file 1 [file Data_Sheet_1.docx]

**Table S1. List of genotypes used for initial screening in a pot experiment and their visual performance under water stress**

| **Sr. No.** | **Genotypes** | **Source** | **SES Scoring** | **Tolerance level** |
| --- | --- | --- | --- | --- |
| 1 | Super Basmati (Susceptible check variety) | Pakistan | 9 | HS |
| 2 | IR-55419-04 (Tolerant check variety) | IRRI | 1 | T |
| 3 | NIBGE-DB-01 | Pakistan | 3 | MT |
| 4 | NIBGE-DB-02 | Pakistan | 5 | MS |
| 5 | NIBGE-DB-03 | Pakistan | 3 | MT |
| 6 | NIBGE-DB-04 | Pakistan | 5 | MS |
| 7 | NIBGE-DB-05 | Pakistan | 3 | MT |
| 8 | NIBGE-DT-02 | Pakistan | 1 | T |
| 9 | NIBGE-DB-06 | Pakistan | 3 | MT |
| 10 | NIBGE-DB-07 | Pakistan | 5 | MS |
| 11 | NIBGE-DB-08 | Pakistan | 7 | S |
| 12 | NIBGE-DB-09 | Pakistan | 3 | MT |
| 13 | NIBGE-DB-10 | Pakistan | 3 | MT |
| 14 | NIBGE-DB-11 | Pakistan | 9 | HS |
| 15 | NIBGE-DB-12 | Pakistan | 3 | MT |
| 16 | NIBGE-DB-13 | Pakistan | 3 | MT |
| 17 | NIBGE-DB-14 | Pakistan | 5 | MS |
| 18 | NIBGE-DB-15 | Pakistan | 7 | S |
| 19 | NIBGE-DB-16 | Pakistan | 7 | S |
| 20 | NIBGE-DT-11 | Pakistan | 1 | T |
| 21 | KSK-133 | Pakistan | 1 | T |
| 22 | NIBGE-DB-17 | Pakistan | 7 | S |
| 23 | NIBGE-DB-19 | Pakistan | 3 | MT |
| 24 | NIBGE-DB-20 | Pakistan | 7 | S |
| 25 | NIBGE-DB-21 | Pakistan | 5 | MS |
| 26 | NIBGE-DB-22 | Pakistan | 7 | S |
| 27 | NIBGE-DB-23 | Pakistan | 9 | HS |
| 28 | NIBGE-BR-18 | Pakistan | 9 | HS |

**Super Basmati:** Drought susceptible check variety, **IR-55419-04:** Drought tolerant check variety, **IRRI**, International Rice Research Institute; **NIBGE,** National Institute for Biotechnology and Genetic Engineering; **SES,** Standard Evaluation Scoring system for rice; **HT,** Highly tolerant; **T,** tolerant; **MT,** moderately tolerant; **MS,** moderately susceptible; **S,** susceptible; **HS,** highly susceptible

**Table S2. Drought Standard evaluation scoring System for rice (IRRI,2014)**

| **Trait** | **Score** | **Description** |
| --- | --- | --- |
| **Drought Tolerance Score** | 0 | Highly tolerant (HT): No symptoms |
|  | 1 | Tolerant (T): Slight tip drying |
|  | 3 | Moderately tolerant (MT): tip drying extended up to ¼ length in most of the leaves |
|  | 5 | Moderately susceptible (MS): 1/4^th^ to ½ of leaves dried |
|  | 7 | Susceptible (S): more than 2/3^rd^ of all leaves fully dried |
|  | 9 | Highly susceptible (HS): All plants apparently dead. Full length in most of the leaves fully dried |

**Table S3. Selected genotypes for seedling stage experiment under hydroponic conditions**

| **Sr. No.** | **Genotypes** | **Tolerance level** |
| --- | --- | --- |
| 1 | IR-55419-04 | Tolerant |
| 2 | Super Basmati (SB) | Susceptible |
| 3 | NIBGE-DT-02 | Tolerant |
| 4 | NIBGE-DT-11 | Tolerant |
| 5 | KSK-133 | Tolerant |
