# Supplementary figures and images for: Integrated Analysis of Osmotic Stress and Infrared Thermal Imaging for the Selection of Resilient Rice Under Water Scarcity
Source: Front Plant Sci. 2022 Feb 14;13:834520. doi: 10.3389/fpls.2022.834520 (PMC8882677; doi:10.3389/fpls.2022.834520)

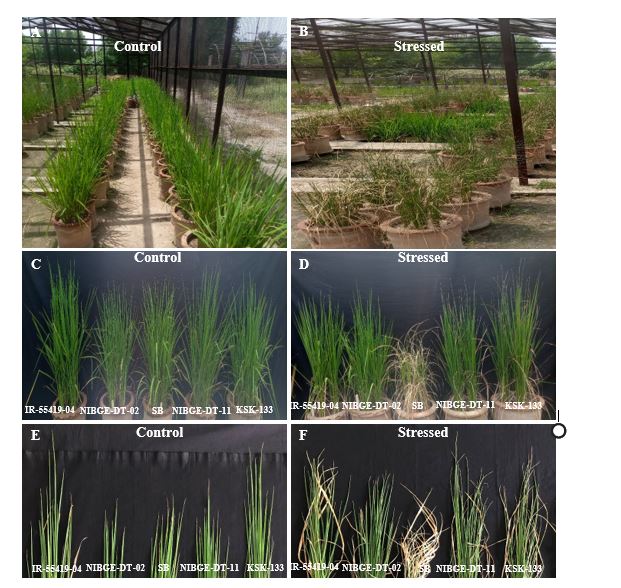

Supplement: Supplementary Figure 1 — Initial screening of rice genotypes under net house conditions. (A) Control-well irrigated rice plants till 30 days after transplantation (DAT). (B) Stressed-water stress rice plants (water withholding for 15 days after 30 DAT) under net house conditions. (C) Control-well irrigated five rice genotypes viz, IR-55419-04, NIBGE-DT-02, SB, NIBGE-DT-11, and KSK-133 selected from initial screening. (D) Stressed-water stress five rice genotypes viz, IR-55419-04, NIBGE-DT-02, SB, NIBGE-DT-11, and KSK-133. (E) Control-leaves of plants of genotypes IR-55419-04, NIBGE-DT-02, SB, NIBGE-DT-11, and KSK-133, respectively, under well water conditions. (F) Stressed- leaves of plants of genotypes IR-55419-04, NIBGE-DT-02, SB, NIBGE-DT-11 and KSK-133, respectively, under water deficit conditions. [file Image_1.JPEG]
